# Analysis of the Spanish Auditory Test of Speech in Noise (PAHRE) in a Population with Hearing Loss

**DOI:** 10.3390/audiolres14050073

**Published:** 2024-09-25

**Authors:** Marlene Rodríguez-Ferreiro, Montserrat Durán-Bouza, Victoria Marrero-Aguiar

**Affiliations:** 1 Psychology Department, University of A Coruña, 15071 A Coruña, Spain; marlene.rodriguez@udc.es; 2 Spanish Language and General Linguistics Department, National University of Distance Education (U.N.E.D.), 28040 Madrid, Spain; vmarrero@flog.uned.es

**Keywords:** speech in noise, hearing loss, hearing test in Spanish

## Abstract

Background: Speech recognition in noise is one of the difficulties faced by people with hearing loss that increases with age. The recently developed Spanish Auditory Test of Speech in Noise (Prueba Auditiva de Habla en Ruido en Español, PAHRE) allows for the identification of these intelligibility difficulties in noise. The aim of this study was to assess speech recognition in noise in people with hearing loss and to test the benefits of the Lombard effect. Methods: The participants were 104 people with hearing difficulties, ranging in age from 37 to 98 years. The variables age, degree of hearing loss, presence of high-frequency dropout, and years of formal education were assessed. Results: Psychometric curves were obtained as a function of signal-to-noise ratio as well as threshold values of speech reception as a function of age group for mild and moderate hearing loss. The results indicated that the speech reception threshold increased with both age and the degree of hearing loss, becoming particularly significant after the age of 70. Furthermore, it was found that the combined factors of age, degree of hearing loss, and educational level predicted a high percentage of the variability in the speech reception threshold. Conclusions: Therefore, the Spanish Auditory Test of Speech in Noise could be a useful clinical tool for diagnosis, monitoring, auditory rehabilitation planning, and hearing aid fitting.

## 1. Introduction

Research has shown that the likelihood of hearing loss increases with age and is directly related to a decrease in speech understanding, especially in noisy environments [1,2]. This difficulty in speech recognition based on the speech-in-noise ratio (SNR) is closely linked to cognitive performance [3]. However, it is important to consider that the variables related to hearing loss (degree, type, cause, duration, etc.) exhibit significant individual variability, resulting in wide variations in speech recognition in noise among individuals with hearing loss. Even with similar audiograms, speech perception in noise can vary unpredictably [4,5,6].

Presbycusis, or age-related hearing loss, encompasses dysfunctions in peripheral hearing, auditory pathway, and cortical organisation. It involves a reduction in cochlear nonlinearity, changes in spectral resolution, and deficits in temporal processing [7,8]. The neurological basis includes decreased functional connectivity in the afferent auditory pathways, which affects the encoding of the temporal properties of the envelope and the fine structure of speech. Additionally, age-related cognitive impairments contribute to the decline in speech discrimination in challenging environments [9,10]. Therefore, Lentz et al. [4] emphasize the importance of considering age-related cognitive impairments when interpreting differences in performance on psychoacoustic tasks between age groups. These impairments are independent of audiometric thresholds [11,12,13].

The auditory system has mechanisms to cope with the difficulties of recognizing speech in noise and improving intelligibility. Some of these mechanisms stem from the signal itself and are realized through bottom–up processing flows, such as spatial release from masking that has been attributed to the effects of head-shadow and binaural processing that analyses the differences in the simultaneously occurring binaural target and masking signals [14,15,16]. Others derive from cognitive resources and are realized through top–down processing, such as the perceptual restoration of lost fragments in the speech signal [17].

Perceptual restoration of missing fragments in the speech signal enhances performance in auditory tasks by utilizing context and prior knowledge [18,19,20]. Context can provide redundant information on several levels: subphonological, phonological, grammatical, lexical-semantic, and pragmatic [21,22]. Older adults can effectively balance the contributions of bottom-up and top-down processes. However, this process is cognitively demanding and varies according to hearing loss or age, affecting a range of both linguistic and non-linguistic processes [19,20,21,22,23,24].

It is important to note that verbal communication involves interaction between the sender and receiver. The speaker unconsciously adapts to noise by making modifications to the speech to improve message intelligibility for the receiver. This adjustment goes beyond increasing signal intensity, involving an increase in the fundamental frequency, the frequencies of the first two formants, and a general upward shift in the speech spectrum, where noise energy is lower. Additionally, there is an increase in vowel duration and intensity, as well as syllable duration, accompanied by more pronounced facial movements. This phenomenon, known as the Lombard effect, has been confirmed by numerous studies for its positive impact on improving intelligibility (for a review, see [25]).

In summary, the mechanisms that allow us to cope with the effects of noise, whether through bottom–up or top–down processing, may be impaired due to deficits associated with hearing loss and/or age, contributing to a reduced ability to understand speech in these situations. However, the role of the Lombard effect may persist throughout life. Although speech discrimination tends to be generally worse in older people, it may still improve when the signal has been adapted through all these noise adaptation mechanisms.

The various factors influencing speech performance in noisy environments underscore the need for effective auditory measures to assess speech perception in such contexts. Regarding supra-threshold measures, tests such as spectro-temporal modulation detection performance and temporal resolution, or more commonly, speech-in-noise tests, are used [26,27]. However, there is a lack of uniformity among the data from different studies regarding the range of SNR to be tested in order to estimate the speech reception threshold (SRT). Killion [28] defines the SNR loss as the difference (in dB SNR) between a given hearing-impaired listener’s 50% correct SNR and the average normal hearing listener’s 50% correct SNR. For example, Wilson et al. [29] indicate that an SNR of 8–10 dB is needed for subjects with hearing loss compared to 1–4 dB for subjects with normal hearing, a difference of approximately 6 dB. Billings and Madsen [30] show a loss effect varying between 2 and 12 dB. Dillon [31] estimates an additional SNR of 1–3 dB for every 10 dB of hearing loss. More recently, Aghasoleimani et al. [32] quantified a difference of 2.34 dB between moderate and severe hearing loss.

This study had two main objectives. The first was to determine whether the PAHRE test is effective for assessing SNR loss, considering age, degree of hearing loss, years of formal education, and high-frequency hearing decline. The second objective was to evaluate whether incorporating the Lombard effect into the test provides an advantage for the audiological assessment of speech in noise.

## 2. Method

### 2.1. Participants

Participants were recruited through a variety of channels, including social media, email, posters, and word of mouth. Participation was voluntary and unpaid, requiring participants to sign an informed consent form after understanding the purpose and objectives of the study.

Inclusion criteria included being over 18 years of age, having hearing loss, and having no impairment in speech production. The final sample consisted of 104 people (41 women and 63 men) with a mean age of 68.34 years (range 37–98). Participants were classified according to age groups and characteristics of hearing loss, such as degree and presence of high-frequency drop-off. The 104 participants had symmetrical hearing loss in terms of type, degree, and configuration of the audiometric curve, as well as discrimination in the disyllabic word test.

The age distribution comprised five groups: 18–49 years (11 participants), 50–59 years (12 participants), 60–69 years (29 participants), 70–79 years (35 participants), and over 80 years (17 participants). Regarding hearing loss, 38 participants had mild hearing loss, 58 had moderate hearing loss, and 8 had severe–profound hearing loss. Of the total sample, 81 exhibited hearing loss above 2000 Hz or had higher hearing thresholds above this frequency compared to frequencies below 2000 Hz.

Additionally, participants were categorized according to their educational level: 55 participants had 10 or fewer years of formal education, 29 had 11–12 years of formal education, and 20 had 13 or more years of formal education. The socio-demographic characteristics of the sample according to age levels and degree of hearing loss are shown in Table 1.

### 2.2. Instruments

Personal and clinical data were obtained from the audiological history. Standard hearing tests were performed to assess hearing loss in terms of symmetry, type, degree, and configuration. Tests included otoscopy, pure tone audiometry, discomfort threshold, and verbal audiometry with disyllabic words [33]. The classification of hearing loss was based on the 500, 1000, 2000, and 4000 Hz octaves [34].

Speech discrimination in noise was assessed using the PAHRE test [25], which consists of 16 blocks with two lists of sentences each: one without the Lombard effect and the other with the Lombard effect. To induce the Lombard effect, multitalker babble noise was presented to the speaker through inserted headphones via an app, calibrated using the “live listening” function to a level of 78 dB SPL. Multitalker noise was chosen because it is more effective in eliciting the Lombard effect due to its shared spectral components with vocalization. The noise consisted of multiple speakers, specifically three women and one man. The speaker was instructed to read the sentences as they had during the previous recording without headphones. The 78 dB SPL intensity was selected to effectively evoke the Lombard effect without causing excessive, exaggerated, or unnatural hyper-articulation. Speech was recorded using a Gefell M930 cardioid condenser microphone (Microtech Gefell GmbH, Gefell, Germany) positioned approximately 30 cm from the speaker, pre-amplified with a JZ Track, and digitized at 44.1 kHz and a 16 bit resolution using an Avid HD-Omni interface (Avid Technology, Burlington, United States) with Avid Protools 2021.3 software.

Each list had six sentences containing five keywords each that were presented with different SNRs (+12, +6, +3, 0, −3, −6 dB). The background noise used produced an energetic and informative masking, as has been demonstrated in a normal-hearing and hearing-impaired population. It is a babble generated by three women and one man, each reading a balanced text, recorded individually and as a group, with the four individual recordings superimposed on the group recording. The recording equipment used was the Alesis Multimix 16 USB (inMusicBrands, Hampshire, United Kingdom) and an AKG C2000B table microphone (AKG Acoustics, Northridge, United States). The recording software used was Adobe Audition 1.0. The recording was made in mono, with a sampling frequency of 44,100 Hz and a resolution of 16 bits. The average noise intensity measured by Praat software was 66.8 dB, with a maximum of 70.4 dB, a minimum of 62.5 dB, and a standard deviation of 1.28 dB [35]. By example, two of the phrases used were as follows:

[Mis tíos empezaron la reforma de la casa familiar] My uncles started the renovation of the family home.

[El líder concluyó la etapa de cien kilómetros] The leader completed the 100-km stage.

The test had high internal consistency reliability for both lists (α = 0.86 and α = 0.89) [25]. Testing was conducted in an acoustically conditioned room and presented through TDH 39 headphones using an Equinox 2.0 clinical audiometer (Interacoustics, Middelfart, Denmark).

### 2.3. Procedure

After completing the clinical history, a hearing assessment was conducted using a battery of tests lasting approximately 30 min. Subsequently, a speech in noise using the PAHRE test was performed, with a duration of approximately 3 min for the presentation of one of the test blocks, resulting in a total of 12 sentences. The presentation intensity of the target speech was adjusted following QuickSIN recommendations [36], aiming for a level above the comfortable threshold for perceiving verbal material. This level coincided with the intensity that provided maximum intelligibility in the initial discrimination test using disyllabic words. The intensity ranged from 20 to 35 dB above threshold, depending primarily on the results of the prior disyllabic word test and, secondarily, on the subject’s feedback. This approach ensures signal comfort without compromising audibility.

Participants were asked to repeat the sentences they heard, with one point awarded for each correct word. Responses were recorded by noting the number of keywords correctly repeated in each sentence for each SNR and the total number of words in the six sentences. SRT was calculated using the Spearman–Kärber equation [37].

Pauses were incorporated between tests to prevent inattention and auditory fatigue. 

The research was approved by the Ethics Committee of the University of A Coruña (UDC) under file number 2020-0027.

### 2.4. Data Analysis

The statistical analysis was conducted using SPSS version 28.0. A descriptive analysis of the scores was performed for each SNR and SRT, both without and with the Lombard effect, considering the degree of hearing loss, age group, high-frequency drop-off, and years of formal education. SRT scores were calculated using the Spearman–Kärber method, representing the level at which 50% of the presented verbal messages were correctly repeated. The formula used was as follows: SRT = *i* + ½(*d*) − d(*r*)/(*n*), where *i* is the initial presentation level, *d* the step size between each SNR, *r* is the total score, and *n* is the number of keywords per sentence [35].

A univariate Analysis of Variance (ANOVA) was applied to investigate differences in SRT scores based on age, degree of hearing loss, years of formal education, and presence of high-frequency drop-off. To assess the extent to which these four variables could predict speech recognition in noise, a stepwise linear regression analysis was performed.

Finally, differences between SRT values obtained in the lists without and with the Lombard effect were analysed using a *t*-test for related samples.

## 3. Results

After administering standard audiological tests to assess hearing loss, the verbal audiometry results with disyllabic words confirmed that all participants had audiometric curves that ruled out any retrocochlear component associated with their hearing loss. Additionally, the audiometry results indicated that all participants exhibited sensorineural hearing loss.

Analyses of the PAHRE data were carried out separately for the results obtained from the lists spoken in silence (without the Lombard effect) and those that were recorded while the speaker was receiving multi-speaker noise through headphones (with the Lombard effect).

### 3.1. Results for the Lists Spoken in Silence

A descriptive analysis of the SRT scores obtained by the participants was conducted, considering variables such as age group, degree of hearing loss, years of schooling, and high-frequency drop-off (see Table 2).

Normality tests using Shapiro–Wilk and Kolmogorov–Smirnov for the variables age, degree of hearing loss, years of formal education, and high-frequency drop-off were not statistically significant. Therefore, a univariate ANOVA was conducted to test for significant differences in SRT scores based on these variables. The ANOVA results revealed significant differences for all variables, except for high-frequency drop-off: age group (*F*(1, 4) = 12.42, *p* ≤ 0.001); degree of hearing loss (*F*(1, 2) = 8.8, *p* ≤ 0.001); and years of formal education (*F*(1, 2) = 15.86, *p* ≤ 0.001).

To explore the relationship between SRT scores and the four variables of interest, a Pearson correlation analysis was performed. The detailed results are as follows:

#### 3.1.1. Age

SRT scores increased with age, with a difference of 5.64 dB between the youngest and oldest participants. The most pronounced increase in SRT scores was observed between the 70–79 and 80+ age groups, with a difference of 1.83 dB. Notably, the largest deviation also occurred in these two age groups.

Post hoc analyses using the Bonferroni test revealed significant differences in SRT scores between the youngest group and all groups aged 60 years and older (*p* = 0.01, *p* ≤ 0.001, *p* ≤ 0.001, respectively). For the 50–59 age group, significant differences were observed with the two older age groups (*p* = 0.015, *p* ≤ 0.001, respectively). Significant differences were also found in the 60–69 age group compared to the younger group *(p* = 0.01) and the older age group (*p* = 0.002). The 70–79 age group showed differences compared to the two younger age groups (*p* ≤ 0.001, *p* = 0.015, respectively). Finally, the group aged 80+ years differed from all groups under 70 years (*p* ≤ 0.001, *p* ≤ 0.001, *p =* 0.002, respectively).

Pearson’s correlation coefficient indicated a positive correlation with age (*r* = 0.57, *p* ≤ 0.001).

#### 3.1.2. Hearing Loss

SRT scores increased with the degree of hearing loss, with a difference of 3.7 dB between the lowest and highest degrees of hearing loss. Differences between adjacent degrees of hearing loss were 1.78 dB between mild and moderate, and 1.92 dB between moderate and severe–profound.

Post hoc analyses revealed statistically significant differences between participants with mild hearing loss and those with moderate and severe–profound hearing loss (*p* = 0.005, *p* = 0.002, respectively). Pearson’s correlation coefficient showed a positive correlation with the degree of hearing loss (*r* = 0.38, *p* ≤ 0.001).

#### 3.1.3. Years of Formal Education

SRT scores decreased as the level of education increased, with the largest deviation in the group with fewer years of schooling. The total difference in SRT scores was up to 2.89 dB, with the most substantial difference of 2.67 dB between those with the fewest years of formal education and the next highest group.

Post hoc analyses using the Bonferroni test revealed significant differences between participants with fewer than 10 years of formal education and those with 11–12 years and 13 or more years of education (*p* ≤ 0.001 in both cases). Pearson’s correlation coefficient showed a negative correlation with years of formal education (*r* = −0.45, *p* ≤ 0.001). 

#### 3.1.4. High-Frequency Hearing Loss

Regarding high-frequency hearing loss, although SRT scores were higher in participants with this condition, the difference compared to those without it was only 0.73 dB. Greater deviations were noted in individuals with flat audiograms or more significant low-frequency losses. Pearson’s correlation coefficient revealed no significant correlation with high-frequency hearing loss.

#### 3.1.5. Age and Hearing Loss

Since hearing loss and age are closely related, and each age group may have different degrees of hearing loss, a descriptive analysis of the SRT scores integrating both variables was performed (see Table 3).

A progressive increase in SRT values was observed with both increasing age and degree of hearing loss, reaffirming the previous results that considered age or degree of hearing loss separately. The most notable differences in SRT scores were found from age 60 onwards for mild hearing loss. For the severe–profound hearing loss, the most significant increase in SRT scores was observed from age 70 onwards, reaching a difference of 5.10 dB. For moderate hearing loss, the increases in SRT scores were gradual with age, with no specific age group showing a sudden increase.

**Figure 1:**
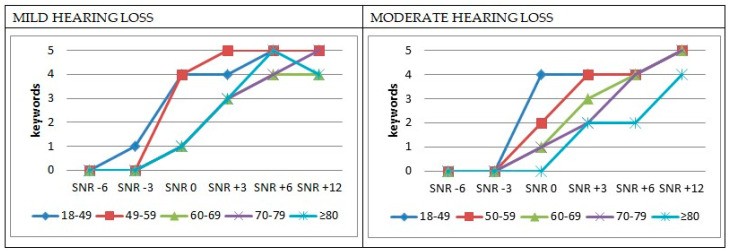
Mean SNR scores by age and degree of hearing for the lists spoken in silence.

The mean SNR scores obtained in the PAHRE test, categorized by age group and degree of hearing loss, are presented in Figure 1. For clarity, only participants with mild and moderate hearing loss are included in this analysis. Cases of severe–profound hearing loss were excluded due to the small sample size in this category: one participant in the two youngest age groups and two participants in the subsequent age groups.

Word recognition was quite similar for participants aged 60 years and older with mild hearing loss, showing a gradual decline from SNR +12 to SNR −3, where recognition dropped to zero across these three age groups. In contrast, the two younger groups demonstrated high recognition levels, which remained relatively stable up to SNR 0. From this point, a sharp decline was observed: recognition dropped from 4 to 1 or 0 correctly repeated words at SNR −3 in the 18–49 and 50–59 age groups, respectively. Although all age groups exhibited a decline in word recognition as SNR decreased, the rate of this decline varied.

A decline was evident across all age groups as hearing loss progressed from mild to moderate, except in the 60–69 age group. The most significant differences were observed between SNR +3 and 0, though for the oldest age group, this decline occurred starting from SNR +6.

Notably, the two younger groups did not display a progressive decline with decreasing SNR, unlike the older groups. Instead, there was an abrupt drop when moving from 4 to 0 repeated keywords in the 50–59 age group with mild hearing loss and in the 18–49 age group with moderate hearing loss, specifically between SNR 0 and SNR −3. Comparing SRT data with the SRT data from the authors’ study in a young normal-hearing population using the same test [38], it is concluded that SNR loss increases with increasing age and degree of hearing loss, taking as a reference the SRT value of 1.99 dB in the non-Lombard condition. The average SNR loss was 0.54, 0.71, 3.54, 3.83, and 3.91 dB, respectively, for each age group with a mild hearing loss and 3.11, 5.02, 5.89, 6.70, and 8.81 dB with a moderate hearing loss.

Based on these findings, a stepwise regression analysis was conducted to evaluate the extent to which each of the three variables could predict variations in SRT scores. The analysis revealed three statistically significant models. The first model included age (*F*(1, 102) = 50.14, *p* ≤ 0.001), with *R*^2^ = 0.33, indicating that 33% of the changes in SRT scores could be explained by age. The second model included both age and degree of hearing loss (*F*(2, 101) = 34.32, *p* ≤ 0.001), resulting in an increase in *R*^2^ to 0.40, which meant a change in 0.07 in *R*^2^. The third model incorporated age, degree of hearing loss, and years of formal education (*F*(3, 100) = 29.25, *p* ≤ 0.001), increasing the *R*^2^ to 0.47, a change of 0.07 in *R*^2^. The resulting regression equation was 2.16 + 1.00 * (age group) + 1.28 * (hearing loss degree) − 0.98 * (years of formal education).

In summary, while all three variables predict variability in SRT scores, age accounts for the highest percentage of the variance.

### 3.2. Results for Lombard Lists

The statistical analyses for the Lombard lists were conducted similarly to those for the silent lists, beginning with a descriptive analysis of the SRT scores, considering age group, degree of hearing loss, years of formal education, and drop in high frequencies (see Table 4).

The Shapiro–Wilk and Kolmogorov–Smirnov normality tests for the variables age group, degree of hearing loss, and years of formal education were not statistically significant, indicating that parametric tests were appropriate for analyzing differences in SRT scores related to these variables. However, the normality tests for the variable drop in high frequencies were significant (*K-S* (81, 23) = 0.13, *p* = 0.003; *W* (81, 23) = 0.95, *p* = 0.004), necessitating the use of a non-parametric test to assess differences in SRT scores for this variable.

Univariate ANOVA was applied to the variables age group, degree of hearing loss, and years of formal education, revealing statistically significant differences for all three variables: age group (*F*(1, 4) = 12.27, *p* ≤ 0.001); hearing loss degree (*F*(1, 2) = 10.88, *p* ≤ 0.001); years of formal education (*F*(1, 2) = 24.70, *p* ≤ 0.001).

To explore potential relationships between SRT scores and the four variables under study, a Pearson correlation analysis was conducted, with results detailed below.

#### 3.2.1. Age

SRT scores increased with age, showing a total difference of 6.76 dB between the youngest and oldest participants. The most significant increase in SRT values was observed between the 70–79 and 80+ age groups, with a difference of up to 3.11 dB. Notably, the largest deviation also occurred in these two age groups.

Bonferroni post hoc analyses indicated significant differences in SRT scores between the youngest group and those aged 70 years and older (*p =* 0.013 and *p* ≤ 0.001, respectively). For the 50–59 age group, significant differences were noted only with participants aged 80 years and older (*p* ≤ 0.001). In the 60–69 age group, differences were observed compared to the age groups immediately above (*p* = 0.026 and *p* ≤ 0.001, respectively). Participants aged 70–79 years showed significant differences in SRT scores compared to the younger group (*p* = 0.013), the 60–69 age group (*p* = 0.026), and the 80+ age group (*p =* 0.013). The oldest age group exhibited significant differences compared to all other age groups (*p* ≤ 0.001, *p* ≤ 0.001, *p* ≤ 0.001 and *p* = 0.013, respectively). Pearson’s correlation coefficient revealed a positive correlation with age (*r* = 0.53, *p* ≤ 0.001).

#### 3.2.2. Hearing Loss

SRT scores increased with the severity of hearing loss, with a total difference of 4.89 dB between the lowest and highest degrees of hearing loss. The largest difference was observed between mild and moderate hearing loss, with a difference of 2.89 dB.

Post hoc analyses revealed significant differences between participants with mild hearing loss and those with moderate and severe–profound hearing loss (*p ≤* 0.001 in both cases). No significant differences were found between moderate and severe–profound hearing loss groups. Pearson’s correlation coefficient showed a positive correlation with the degree of hearing loss (*r* = 0.42, *p ≤* 0.001).

#### 3.2.3. Years of Formal Education

SRT scores decreased with higher levels of education, showing greater variability in the group with fewer years of schooling. The difference in SRT scores between participants with fewer years of formal education and those with the next highest level was 4.02 dB, increasing to 4.78 dB when compared to those with the most years of education.

Post hoc analyses using the Bonferroni test revealed significant differences between participants with fewer than 10 years of formal education and those with 11–12 and 13 or more years of education (*p* ≤ 0.001 in both cases). Pearson’s correlation coefficient showed a negative correlation with years of formal education (r = −0.54, *p* ≤ 0.001).

#### 3.2.4. High-Frequency Hearing Loss

Although SRT scores were slightly higher for participants with high-frequency hearing loss, the difference compared to those without this condition was only 0.69 dB. Greater deviations were noted in participants with flat audiograms or a significant low-frequency loss. Pearson’s correlation coefficient revealed no significant correlation with high-frequency hearing loss. Mann–Whitney U-tests showed no statistically significant differences between the groups.

#### 3.2.5. Age and Hearing Loss

Given that each age group may display different levels of hearing loss, a descriptive analysis of SRT scores considering both variables was performed (see Table 5). The analysis confirmed a progressive increase in SRT values with both age and degree of hearing loss, supporting the results obtained when these variables were considered separately. Although the increase was evident, it was not linear. The most substantial differences were observed from age 70 for mild and severe–profound hearing loss, and from age 80 for moderate loss, reaching a difference of 3.38 dB compared to the immediately preceding age group.

**Figure 2:**
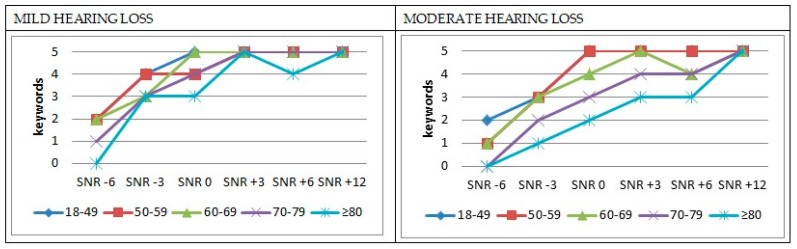
Mean SNR scores by age and hearing loss degree in lists with Lombard effect.

Figure 2 presents the mean SNR scores categorized by age group and degree of hearing loss. Consistent with the silent lists, this analysis includes only participants with mild and moderate degrees of hearing loss, as cases with severe–profound hearing loss were excluded due to their limited representation.

A greater impairment in word recognition was observed with decreasing SNR and increasing age and degree of hearing loss. However, the onset of this decline varied by degree of hearing loss. For mild hearing loss, the scores remained relatively stable across all age groups up to SNR +3, whereas, with moderate hearing loss, differences between age groups became noticeable at SNR +6. The decline in word recognition with decreasing SNR was progressive across all age groups and degrees of hearing loss, except for a notable exception in the oldest age group with mild hearing loss, which showed a sharper drop.

The three youngest age groups exhibited a consistent trend across different SNR levels, whether they had mild or moderate hearing loss, including at the lowest SNR levels. Only the 70–79 age group with moderate hearing loss and the 80+ age group, regardless of hearing loss severity, failed to correctly repeat any key words at SNR −6. Comparing SRT data with the SRT data from the authors’ study in a young normal-hearing population using the same test [38], it is concluded that SNR loss increases with increasing age and degree of hearing loss, taking as a reference the SRT value of −2.95 dB in the Lombard condition. The average SNR loss was 0.85, 1.60, 1.54, 2.89, and 3.85 dB, respectively, for each age group with mild hearing loss and 2.25, 2.74, 3.16, 4.97, and 8.35 dB with moderate hearing loss.

A stepwise regression analysis was conducted to examine the extent to which each of the three variables could predict variations in SRT scores. Three statistically significant models were identified. The first model, which included years of formal education, accounted for 29% of the variance in SRT scores (*F*(1, 102) = 42.48, *p* ≤ 0.001, *R*^2^ = 0.29). The second model, which included both years of formal education and degree of hearing loss, explained 43% of the variance (*F*(1, 101) = 23.21, *p* ≤ 0.001, *R*^2^ = 0.43), reflecting a change of 0.13 in *R*². The third model, incorporating all three variables, accounted for 52% of the variance (*F*(1, 100) = 18.34, *p* ≤ 0.001, *R*^2^ = 0.52), representing a change of 0.09 in *R*^2^. The final regression equation was −2.674 − 1.903(years of formal education) + 1.97(hearing loss degree) + 1.039 * (age group).

In summary, while all three variables predict variability in SRT scores, years of formal education explains the greatest percentage of the variance in the case of the Lombard lists.

## 4. Discussion and Conclusions

As expected, when using a speech-in-noise test, results from the PAHRE test in persons with hearing loss showed an increase in SRT scores with increasing age and degree of hearing loss. The increase in SRT values was progressive, although from the age of 70 years onwards, the increase was more pronounced. These results make it difficult to estimate a mean increase in SRT per decade, as found in other studies [1,2]. The average SRT increase per decade obtained gave values of 0.48 and 0.81 dB for mild and moderate loss, respectively, in the case of spoken silence lists, and 0.43 and 0.87 dB for Lombard lists.

The estimation of the SRT increase per 10 dB loss gave results of 0.56 and 0.49 dB for the 50s and 60s, respectively, in the spoken silence lists and 0.56 and 0.62 dB for the same groups in the Lombard lists. These results align with those obtained by previous studies [29,30,32]. 

Decreasing frequencies above 4000 Hz did not predict changes in SRT in people with hearing loss. Therefore, the increase in SRT is due to a decrease in hearing thresholds, regardless of the slope of the psychometric function.

Although no cognitive tests were performed in this study, the educational level of the participants was considered. This variable is associated with the level of cognitive function before old age and is used as an indicator of cognitive reserve [39,40,41]. The results showed a direct relationship between educational level and SRT, with lower scores associated with increasing years of formal education. Although this variable cannot predict cognitive impairment or replace the need to assess cognitive processes, it could be included as an item in a patient’s clinical history due to its effect on SRT.

Together, these three variables (age, degree of hearing loss, and educational level) showed a strong relationship with SRT, accounting for 46.7% of the variance in the spoken silence lists and 51.5% in the case of the Lombard lists. These results align with those obtained by Lentz et al. [3], which indicate a direct relationship between difficulty in speech recognition in noisy environments, age, hearing loss, and cognitive performance.

In view of these results, there is a clear need to interpret differences in performance in psychoacoustic tasks, such as SIN tests, according to age groups. This is motivated by the cognitive impairments commonly associated with aging, which, together with hearing loss, negatively influence auditory perception skills, especially word recognition in noise [3,42].

### Lombard Effect

The most realistic way to assess SNR loss should consider all the variables that occur in everyday situations where conversations take place in the presence of ambient noise. However, it is difficult to carry out this type of assessment in everyday clinical practice due to multiple influencing variables such as speech, speech source, noise sources, and variable SNR. As a result, most studies are conducted in controlled environments, limiting their representation of everyday performance.

The presentation of voiced sentences in noise, which exhibit the Lombard effect, is of great interest as it resembles a conversation in a real-life situation. The PAHRE test offers the possibility to present these sentences in addition to sentences voiced in silence, as do most SIN tests.

The results confirm that the Lombard effect supports speech recognition in noise. All ages and degrees of hearing loss benefited from Lombard speech, although the largest differences were observed in the 60–69 and 70–79 age groups with mild and moderate hearing loss. The SNR loss was also lower, especially in the older age group, when compared to the SNR loss in the non-Lombard condition.

Although there are differences between lists voiced in silence and in noise, both showed the same trend concerning age, hearing loss, drop in high frequencies, and years of formal education. SRT scores increased in similar proportions with increasing age and degree of hearing loss but decreased with increasing years of formal education, regardless of whether the stimuli were voiced in silence or noise. However, the models obtained for each list differed considerably in terms of the weight contributed by each variable. Therefore, although the trends were the same, the results for both lists should be considered.

Despite the similarities in the results obtained and the absence of verbal material voiced in noise in other SIN tests, we consider it justified to keep both lists in PAHRE. On the one hand, lists voiced in silence allow for comparable SRT values between different tests under the same voicing conditions, facilitating the interpretation of SNR loss values and the corresponding recommendations based on these values. On the other hand, the Lombard lists enable the continuity of speech-in-noise tests in the adult population while maintaining consistency with the PAVER Spanish speech-in-noise test for the paediatric population [43], which includes a Lombard list among the three lists presented to subjects.

Furthermore, hyperarticulate speech produced under the Lombard effect has specific features of great interest for speech recognition in noise that could be assessed in future studies. Finally, the combined duration of both lists is less than four minutes, supporting their inclusion.

The assessment of the cognitive component of PAHRE in older adults is suggested for future studies. It should be noted that the assessment by this test requires memorising a sentence with five key words, thus depending on working memory capacity. According to previous studies [44], there is evidence of a significant association between cognitive function and hearing loss and SRT in subjects over 60 years of age, an association that was not assessed in the present study.

## 5. Conclusions

The results obtained from the PAHRE test highlight the significant influence of variables such as age, degree of hearing loss, and years of formal education on SNR loss. These findings can facilitate the creation of age-specific SNR psychometric functions for mild and moderate SNR losses.

Incorporating a list of Lombard-effect sentences into a speech-in-noise test provides a more realistic assessment of SNR loss. While the results exhibit similar trends concerning age, degree of hearing loss, and educational level for both lists with and without the Lombard effect, the models derived from them differ. Future research should explore the feasibility and benefits of integrating Lombard speech into speech-in-noise tests.

Considering that the PAHRE test delivers valuable insights into speech recognition in Spanish noise within an application time of less than four minutes, it is positioned as an effective clinical tool. Its implementation could be highly beneficial for diagnosis, monitoring, auditory rehabilitation planning, and hearing aid fitting.

## Figures and Tables

**Table 1:** Distribution of participants according to age and degree of hearing loss.

		Age Groups					
		18–49	50–59	60–69	70–79	≥80	Total
Hearing Loss Degree	Mild	7	4	14	10	3	38
	Moderate	3	7	13	23	12	58
	Severe–profound	1	1	2	2	2	8
	Total	11	12	29	35	17	104

**Table 2:** SRT values as a function of age, degree of hearing loss, years of formal education, and high-frequency drop-off for the lists spoken in silence.

SRT for Binaural Condition					
		Mean	Standard Deviation	Maximum	Minimum
Age group	18–49	2.92	1.68	5.10	−0.30
	50–59	4.15	2.28	8.10	1.50
	60–69	5.76	1.91	9.30	0.90
	70–79	6.73	2.59	13.50	0.90
	≥80	8.56	2.91	13.50	3.30
Hearing loss degree	Mild	4.78	2.23	9.30	−0.30
	Moderate	6.56	2.74	11.70	0.90
	Severe–profound	8.48	3.66	13.50	3.90
Years of formal education	≤10	7.36	2.83	13.50	1.50
	11–12	4.69	2.03	8.70	−0.30
	≥13	4.47	2.08	8.10	0.90
High-frequency drop-off	Yes	6.22	2.53	11.70	0.90
	No	5.49	3.73	13.50	−0.30

**Table 3:** SRT values as a function of age and degree of hearing loss for the lists spoken in silence.

SRT for Binaural Condition						
		Age Group				
		18–49	50–59	60–69	70–79	≥80
Hearing loss degree	Mild	2.53	2.70	5.53	5.82	5.90
	Moderate	3.10	5.01	5.88	6.69	8.80
	Severe–profound	5.10	3.90	6.60	11.70	11.10

**Table 4:** SRT values as a function of age, degree of hearing loss, years of formal education, and presence of high frequency dropout for the Lombard lists.

SRT for Binaural Condition					
		Mean	Standard Deviation	Maximum	Minimum
Age group	18–49	−1.77	1.79	1.50	−4.50
	50–59	−0.85	2.35	3.90	−4.50
	60–69	−0.59	2.81	7.50	−4.50
	70–79	1.88	3.80	13.50	−3.30
	≥80	4.99	3.52	12.30	−0.90
Hearing loss degree	Mild	−0.99	1.87	3.30	−3.90
	Moderate	1.90	3.76	9.90	−4.50
	Severe–profound	3.90	6.55	13.50	−3.30
Years of formal education	≤10	3.04	4.00	13.50	−4.50
	11–12	−0.98	1.54	1.50	−3.90
	≥13	−1.74	2.00	2.10	−4.50
High-frequency drop-off	Yes	1.15	3.47	9.90	−4.50
	No	0.46	4.90	13.50	−4.50

**Table 5:** SRT values as a function of age and degree of hearing loss in lists with Lombard effect.

SRT in Binaural Condition						
		Age Group				
		18–49	50–59	60–69	70–79	≥80
Hearing loss degree	Mild	−2.10	−1.35	−1.41	−0.06	0.90
	Moderate	−0.70	−0.21	0.21	2.02	5.40
	Severe–profound	−2.70	−3.30	0.00	9.90	8.70

## Data Availability

https://doi.org/10.6084/m9.figshare.26435059 (accessed on 30 July 2024).
